# Level of adult client satisfaction with clinic flow time and services of an integrated non-communicable disease-HIV testing services clinic in Soweto, South Africa: a cross-sectional study

**DOI:** 10.1186/s12913-020-05256-9

**Published:** 2020-05-11

**Authors:** Kathryn L. Hopkins, Khuthadzo E. Hlongwane, Kennedy Otwombe, Janan Dietrich, Mireille Cheyip, Nompumelelo Khanyile, Tanya Doherty, Glenda E. Gray

**Affiliations:** 1 Perinatal HIV Research Unit, Faculty of Clinical Medicine, University of the Witwatersrand, Chris Hani Baragwanath Academic Hospital, Johannesburg, South Africa; 2 School of Public Health, Faculty of Health Sciences, University of the Witwatersrand, Johannesburg, South Africa; 3 Health Systems Research Unit, South African Medical Research Council, Cape Town, South Africa; 4 Centers for Disease Control and Prevention, Pretoria, South Africa; 5 School of Public Health, University of the Western Cape, Cape Town, South Africa; 6 Office of the President, South African Medical Research Council, Cape Town, South Africa

**Keywords:** HIV/AIDS, HIV prevention, Non-communicable diseases, HIV testing services, Client satisfaction, Healthcare, Comorbidities

## Abstract

**Background:**

While HIV Testing Services (HTS) have increased, many South Africans have not been tested. Non-communicable diseases (NCDs) are the top cause of death worldwide. Integrated NCD-HTS could be a strategy to control both epidemics. Healthcare service strategies depends partially on positive user experience. We investigated client satisfaction of services and clinic flow time of an integrated NCD-HTS clinic.

**Methods:**

This prospective, cross-sectional study evaluated HTS client satisfaction with an HTS clinic at two phases. Phase 1 (February–June 2018) utilised standard HTS services: counsellor-led height/weight/blood pressure measurements, HIV rapid testing, and symptoms screening for sexually transmitted infections/Tuberculosis. Phase 2 (June 2018–March 2019) further integrated counsellor-led obesity screening (body mass index/abdominal circumference measurements), rapid cholesterol/glucose testing; and nurse-led Chlamydia and human papilloma virus (HPV)/cervical cancer screening. Socio-demographics, proportion of repeat clients, clinic flow time, and client survey data (open/closed-ended questions using five-point Likert scale) are reported. Fisher’s exact test, chi-square analysis, and Kruskal Wallis test conducted comparisons. Multiple linear regression determined predictors associated with clinic time. Content thematic analysis was conducted for free response data.

**Results:**

Two hundred eighty-four and three hundred thirty-three participants were from Phase 1 and 2, respectively (*N* = 617). Phase 1 participants were significantly older (median age 36.5 (28.0–43.0) years vs. 31.0 (25.0–40.0) years; *p* = 0.0003), divorced/widowed (6.7%, [*n* = 19/282] vs. 2.4%, [*n* = 8/332]; *p* = 0.0091); had tertiary education (27.9%, [*n* = 79/283] vs. 20.1%, [*n* = 67/333]; *p* = 0.0234); and less female (53.9%, [*n* = 153/284] vs 67.6%, [*n* = 225/333]; *p* = 0.0005), compared to Phase 2. Phase 2 had 10.2% repeat clients (*n* = 34/333), and 97.9% (*n* = 320/327) were ‘*very satisfied’* with integrated NCD-HTS, despite standard HTS having significantly shorter median time for counsellor-led HTS (36.5, interquartile range [IQR]: 31.0–45.0 vs. 41.5, IQR: 35.0–51.0; *p* < 0.0001). Phase 2 associations with longer clinic time were clients living together/married (est = 6.548; *p* = 0.0467), more tests conducted (est = 3.922; *p* < 0.0001), higher overall satisfaction score (est = 1.210; *p* = 0.0201). Those who matriculated experienced less clinic time (est = − 7.250; *p* = 0.0253).

**Conclusions:**

It is possible to integrate counsellor-led NCD rapid testing into standard HTS within historical HTS timeframes, yielding client satisfaction. Rapid cholesterol/glucose testing should be integrated into standard HTS. Research is required on the impact of cervical cancer/HPV screenings to HTS clinic flow to determine if it could be scaled up within the public sector.

## Background

HIV Testing Services (HTS) (i.e.; pre- and post-test counselling, laboratory services, delivery of results, and the linkage of clients to the required HIV prevention, treatment and care services) has increased in South Africa, with more than 4500 public health facilities offering provider-initiated counselling and testing (PICT) following the launch of the national HTS campaign in 2010 [1, 2]. However, the country is still falling short of the Joint United Nations Programme on HIV/AIDS (UNAIDS) 90–90–90 target, where by 2020, 90% of all people living with HIV (PLHIV) will know their HIV status; 90% of all people with diagnosed HIV infection will receive sustained antiretroviral therapy (ART); and 90% of all people receiving ART will have viral suppression [3]. As of December 2017, only 84.9% of PLHIV aged 15–64 years knew their HIV status (88.9% of HIV-infected females, 78.0% of HIV-infected males), 70.6% of PLHIV were on ART, and 87.5% of PLHIV on ART were virally suppressed (89.9% of females and 82.1% of males) [4]. Although there has been an increase in the uptake in HIV testing (a 19.4% increase since 2012 [5],) a significant number of South Africans have not yet been tested.

Furthermore, the top cause of death and disability worldwide are chronic non-communicable diseases (NCDs), such as cardiovascular disease (CVD) and diabetes mellitus (DM), attributing to more than three in five deaths [6]. In South Africa, cervical cancer, largely caused by the sexually transmitted Human Papilloma Virus (HPV), is the second most prevalent form of (preventable) cancer [7–9]. Lower and middle income countries (LMICs), such as South Africa, are being disproportionately impacted by these diseases [10]. The recent South African Demographics and Health Survey (SADHS) reports alarming levels of unknown NCDs within the population [11]. With the scale-up of ART, HIV is becoming a chronic disease with PLHIV reaching nearly the same life expectancy as healthy individuals [12]. Historically, NCDs are thought of as ‘aging diseases’ [13]. Not only is HIV-infection seemingly associated with an accelerated ‘aging’ process, but PLHIV, now living longer while on treatment, are becoming increasingly at-risk for non-HIV-related chronic conditions of aging similar to the HIV-uninfected population [14].

There are varying models of HTS service provision under investigation to determine the effect on uptake of HIV and/or NCD testing (e.g.; home-based or work-place testing, client-initiated or provider-initiated testing, provision of integrated services, etc.) [15–21]. National and global policies stress integrated screening to address NCD pre-cursors (e.g. blood pressure [BP] and body mass index [BMI]) [22–24]. However, integrated screening is implemented infrequently and/or in a non-standardised manner in many South African HTS, and health programmes require evidence to inform widespread adoption. While integration of HIV with other programmes, such as reproductive health and tuberculosis (TB), has occurred through the establishment of inclusive national policies, both research and evidence-based data on the acceptability, effectiveness, feasibility and sustainability of HIV-NCD integration in clinical practice is scarce [25–27].

While integration of screening, prevention and referral services may be an intuitive solution for combating the morbidity and mortality associated with HIV and NCDs, there is minimal evidence regarding the patient- and service-level effect of such a strategy [28]. Competent care and health systems are necessary for achieving high-quality care. However, it is believed that health systems – and its interventions - be evaluated in part by the confidence of people in their health system and the processes of care, inclusive of assessing user experience [29]. Not only is such measurement critical for accountability and improvement, but a positive user experience has a fundamental value which can improve retention in care, adherence to treatments, and confidence in health systems [29]. Additionally, some studies have found that positive user experience is linked to better technical quality [29], and although patients cannot force the provision of better care, they can improve their health by actively choosing to seek better providers and/or remain in care [30]. The integration of new services could have substantial impacts on clinic operations, provider responsibilities, and the quality of care delivered, causing dissatisfaction among clients [28]. For example, the integration of HIV services with sexual and reproductive health services could result in an increased patient burden and therefore inadequate staffing to support this shift, yielding patient dissatisfaction [31].

The utility of assessing patient/client satisfaction measures, specifically when paired with open-ended responses, is that it gives the recipients of healthcare a voice, which may reveal elements of the quality of the processes of care shaping the user experience [29]. Client ratings and recommendations are critical for accountability and improvement [29], helping to shape service delivery in real-time. There is currently a gap in literature surrounding health system user experience among LMIC [29].

Acceptance of any service – or model of service - is partially dependent on client satisfaction with the service provided. This model focuses on whether client expectations are ‘confirmed’ (i.e.; satisfied) or ‘disconfirmed’ (i.e.; dissatisfied) [32]. Health care is no different [33]. Client-related factors linked to satisfaction in relation to health care may be related to the distance and expense incurred in traveling to the clinic, level of confidentiality maintained, types of services offered and clients’ decision to accept specific services, duration of services and/or wait time, human relations and/or facility environment, and availability of treatment [32, 34, 35]. Client satisfaction, when included as part of a more holistic evaluation, inclusive of complimentary components such as healthcare provider satisfaction and quality of care, is a critical piece to evaluate a healthcare delivery strategy’s feasibility and acceptability.

The Perinatal HIV Research Unit (PHRU) Zazi HTS Clinic, has provided on-site HIV testing and counselling for adults in Soweto, South Africa, since 2001. Zazi also draws blood for CD4 count testing at a contracted laboratory for HIV-infected clients not yet initiated on treatment, as an indicator of stage of disease. Zazi is currently the only service provider of walk-in, free, accessible HTS supplied to the Chris Hani Baragwanath Academic Hospital (Bara Hospital), the largest hospital in the southern hemisphere. HIV treatment is not provided on-site, but clients are given referral letters to local clinics [36].

To reflect the South African National Department of Health’s (NDoH) newly proposed HTS standard of care [22], the Zazi clinic expanded its health service provision in February 2018, and began offering counsellor-led height and weight measurements, BP readings, and symptom screenings for sexually transmitted infections (STIs) and TB within its HIV rapid testing service [36]. As part of a larger feasibility and acceptability study, this standard of care HTS clinic (Phase 1) introduced additional health screening services in June 2018, transforming itself into an integrated NCD-HTS clinic model (Phase 2). The integrated NCD-HTS model included the following services in addition to the standard of care HTS: counsellor-led waist circumference measurements and BMI calculations; rapid blood cholesterol and blood glucose (both random and average [HbA1c]); and for female clients, nurse-led rapid Chlamydia testing, Human Papilloma Virus (HPV) genotyping, and cervical cancer screening via Pap smear.

There is a paucity of client satisfaction research for different integrated service models within scientific literature. The aim of our study was to describe client satisfaction with the services of and clinic flow time within an integrated NCD-HTS clinic in Soweto, South Africa. This paper presents the client clinic exit survey data and clinic flow time collected on each clinic model – standard of care HTS versus integrated NCD-HTS – along with the respective client demographics. We also report the proportion of repeat clients. Understanding client opinions and level of satisfaction regarding each model of health service will provide a deeper understanding of whether or not a particular model is meeting the community’s needs and expectations. Feedback may improve healthcare service and the overall client experience. This study is but one piece of an overall evaluation being conducted on this integrated NCD-HTS model to determine its feasibility and acceptability. Other complimentary components being investigated with results presented elsewhere are HTS provider satisfaction, quality of task-shifting NCD-precursor rapid testing to lay counsellors, and health outcomes (i.e.; referrals, linkage to care and treatment initiation) for HTS clients.

## Methods

### Study design and setting

This is a prospective, mixed-methods study using cross-sectional data evaluating client satisfaction of two phases of a health screening programme within the Zazi HTS centre at PHRU, a leading research centre situated at Bara Hospital in Soweto, South Africa. In Phase 1, the clinic operated under the standard of care HTS guidelines for 3 months (from 19 February and 14 June, 2018). In Phase 2 (between 18 June 2018 and 28 March 2019), the clinic conducted integrated NCD-HTS screening for 9 months. Each phase had its own, independent walk-in client population (i.e.; data were not longitudinal).

### Clinic staffing

The Zazi clinic is unique from government clinics in that it is driven by three counsellors and supported by one nurse, with clients only being triaged to the nurse for specialised endocervical screening procedures requested and/or for any prevention, treatment and/or care referrals required. The staff also catered to a client’s emotional needs and did not limit the time of a counselling session.

### Sampling

Our sampling approach was serial sampling, allowing for all eligible walk-in PHRU Zazi HTS clients who consented to the health screening programme to be approached to partake in a clinic visit exit survey prior to leaving the clinic (a specific informed consent form and survey was used for each Phase) and for their data to be captured and used in research. Consenting clients were enrolled as study participants. Survey data was reviewed and analysed monthly, using chronologically collected surveys, and the sample was deemed complete once content saturation was reached. Further details on recruitment and sampling can be seen in Supplementary Figure 1.

### Inclusion and exclusion criteria

Eligibility criteria for participation in the study were the following: at least 18 years of age; able to communicate in either English, IsiZulu and/or Sotho; and able and willing to provide written or verbal informed consent (with an impartial witness) for health screening procedures and completion of client exit survey. If a client presented to the clinic critically ill, they were not enrolled but referred to the Bara Hospital casualty ward.

### Data collection and management

Client demographic and clinic exit survey data were self-reported and collected either by self-completion of a survey by English-literate clients in the reception area, or collected with the help of the clinic receptionist or lay counsellors for clients unable to read or write. Each survey was checked by clinic staff for logical completion. Clinic flow timeframes from registration through the counsellor-led HTS session were noted and logged on paper forms by either the clinic receptionist, lay counsellors, or the nurse. All surveys and forms comprised part of the PHRU Zazi HTS client file. Data from patient files were cleaned and verified prior to entry into the Research Electronic Data Capture (REDCap) database.

### Study measures

#### Demographic information and repeat clients

Data were collected on sex, age, race, nationality, ethnic group, marital status, highest level of education, employment, and details regarding socio-economic status (type of housing, source of water and fuel for cooking and lighting, type of toilet facility, as well as possession of household and ownership items).

We determined the proportion of Phase 2 clients who were repeat clients within Phase 2.

#### Clinic flow time

**Fig. 1:**
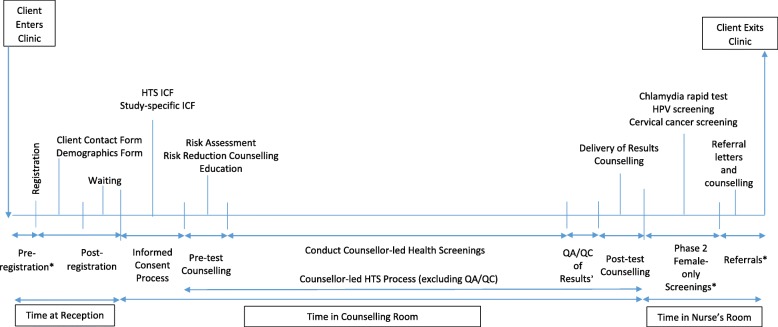
Client clinic flow diagram. Original diagram. Hopkins KL 2020. *Time data not collected

Clinic flow time start and stop time measures (in minutes) were recorded by clinic staff (receptionist for post-registration timeframe, and counsellors for time spent in counselling room) providers in real-time on clinic records (not study-specific records) after direct observation of a computer’s clock Clinic flow is depicted within Fig. 1. Pre-registration time, time taken for QA/QC of results, and time spent within the nurses room (for Phase 2 female-only screenings and provision of referral letters and additional counselling) was not reported. Independent sections of the clinic flow for which time was reported are defined as follows:

- *Time spent at reception, post-registration:* From when a client was registered with the receptionist until the client was received by the counsellor. The time was spent in the waiting room, where the Demographics and Client Contact forms were also completed.
- *For pre-test counselling:* From when the counsellor initiated pre-test counselling until it was concluded.
- *Conducting health screenings:* From when the pre-test counselling concluded until both health screenings and the Family History and Behavioural Questionnaire were completed. The following health screenings were conducted by the counsellor – height/weight measurements, BP readings, STI and TB symptoms screening and HIV rapid testing (for standard of care HTS); and standard of care HTS combined with abdominal girth measurements, BMI categorizations, blood sugar rapid tests (both HbA1c and random), blood cholesterol rapid test (for integrated NCD-HTS). The questionnaire, which was administered while waiting for the finger prick rapid testing results, included symptom screening questions, among others.
- *For post-test counselling*: From when the counsellor initiated post-test counselling until it was concluded. This also included risk-reduction counselling and delivery of all aforementioned counsellor-led health screening results.
- *Total time for counsellor-led HTS process:* From when the pre-test counselling started until post-test counselling ended, excluding the QA/QC of results.

#### Client exit survey

We developed a structured client exit survey which consisted of both close-ended and open-ended questions for free responses to assess client satisfaction with each clinic model. This survey was solely created for the purposes of this study and has not been previously published. The close-ended questions included a five-point Likert format with differing responses for various questions (e.g.; *I feel very satisfied, I feel somewhat satisfied, I feel neither satisfied nor dissatisfied/neutral, I feel somewhat dissatisfied, I feel very dissatisfied*). The Likert responses were designed to assess client satisfaction with each clinic model’s HTS processes (i.e.; pre- and post-test counselling, and preparedness for and level of comfort/discomfort during screening procedures), the helpfulness of speaking with the nurse, and overall satisfaction of each clinic model. Time satisfaction was also assessed throughout the clinic flow.

The responses to the open-ended questions helped us to further understand the responses to the close-ended questions. The open-ended questions included: *Please explain why you opted out of any offered services?; In your own opinion, what improvement(s) should be made for pre-test counselling / for the health screening testing procedure(s)?; In your own words, please describe what you liked / disliked about Zazi today; In your own words, please share if there is anything else you wished the Clinic would have provided you.*

Overall satisfaction score was defined as the sum of scores from the Likert scale with values ranging from 1 to 5 and higher values signifying more satisfaction. The questions included rating the amount of time it took before seeing a counsellor; the amount of time it took the counsellor to provide pre-test counselling, health screenings, and provide results and conduct post-test counselling; and the amount of time spent with the nurse for further consultation and referrals.

### Quantitative data analysis

Frequencies and their percentages were determined for categorical variables and stratified by model of HTS.

To test statistical significance for categorical measures stratified by phase, Fisher’s exact test or chi-square analysis was used, where appropriate. Descriptive statistics, such as medians and interquartile ranges, were determined for continuous measures (e.g.; age) and compared by HTS model non-parametrically using the Kruskal Wallis test. Multiple linear regression was used to determine predictors associated with time spent (in minutes) in the clinic. Regression residuals, including plots, were used to assess model fit.

A wealth quintile was developed using the socio-economic measures and determined using Principal Component Analysis (PCA) [37–39]. The socio-economic measures included *type of house* (shack/zozo [a common isiZulu term meaning *tin-roofed shack*], flat/cottage, room/garage, hostel, shared house, standalone brick house or other); *source of water* (tap water indoors, only outside tap water, well/borehole, stream/river/pond or other); *type of toilet facility* (flush toilet inside [connected to sewage], outside pit latrine/toilet, flush toilet inside [septic tank], no toilet facility or other); *type of fuel used for cooking* (electricity, gas, paraffin, coal, candle, firewood/straw or other); *type of fuel used for lighting* (electricity, gas, paraffin, coal, candle, firewood/straw or other); *possession of household items* such as radio, computer, landline telephone, TV, refrigerator, mobile phone; and ownership of bicycle, car/truck, sheep/goat/cattle, motorcycle/scooter or donkey/horse. Prior to calculating the wealth quintile, variables with more than two levels were recoded into binary (0 or 1) for each level of response. Frequencies were run on all the binary variables, and only those with responses between 5 and 95% were retained. The retained variables were evaluated by the PCA method using the varimax rotation to identify those contributing largely to the wealth construct. Variables meeting a pre-specified cut-off and loading in at least two factors were excluded. The remaining variables were retained and scored to create the socio-economic index. Five wealth quintiles were estimated on the scored measure using 20 percentile intervals, increasing in levels of wealth (i.e.; first quintile was lowest level of wealth and fifth quintile was highest). Client satisfaction results were presented graphically for health screenings.

All statistical analysis was conducted in SAS Enterprise Guide 7.1 (SAS Institute, Cary NC) using the SAS/STAT procedures PROC FREQ, MEANS, NPAR1WAY, FACTOR, SGPANEL, and PROC GLM.

### Qualitative data analysis

Using SAS Enterprise Guide 7.1 (SAS Institute, Cary NC), a print out of all open-ended responses per question was obtained. The first author read through each text response (codes) to gain an overall understanding of the data. All codes were collated in an excel spreadsheet under the pre-determined themes, which were based around quantitative questions. Thereafter the first author developed a codebook by assigning defined categories of grouped codes. Most categories were also pre-determined. However, if a code did not fit nicely, a new category was created. Categories were then analysed further to create more detailed sub-categories.

Content thematic data analysis was utilised by counting the number of codes per category, using SAS. A response to a particular question could be assigned to multiple categories, which may have increase the sample size of certain variables. All non-responses to a particular question (free responses left blank by participants) and responses agreed upon as non-logical were not included in the analysis, thus decreasing the sample size for that question.

Inductive coding was used to deduce meaning from the qualitative data [40]. Concepts from research literature served as deductive codes. Validity of initial codes was checked by identifying whether it is repeated across different responses to the same survey question within the study, highlighted by participants themselves as an important issue [41].

Agreement on the data analysis was achieved through a second review by a co-author.

## Results

### Characteristics of participants and repeat clients

A total of 617 participants of median age 33.0 (IQR: 26.0–41.0) years, were included in the analysis from both HTS models (284 and 333 participants for standard of care HTS and integrated NCD-HTS, respectively), and the largest proportion of participants were females (61.3%, [*n* = 378/617]), black African (99.2%, [*n* = 612/617]), South African (96.6%, [*n* = 592/613]), Zulu speaking (45.3%, [*n* = 279/616]), single (70.7%, [*n* = 434/614]), educated up to high school (40.1%, [*n* = 247/616]), and employed (36.9%, [*n* = 227/615]). More than one quarter were within the third wealth quintile category (i.e.; middle class; 26.6%, [*n* = 164/617]). Participants of standard of care HTS were significantly older (median age was 35.0 (28.0–43.0) years vs. 31.0 (25.0–40.0) years; *p* = 0.0003), likely to be divorced (6.7%, [*n* = 19/282] vs. 2.4%, [*n* = 8/332]; *p* = 0.0091) and be tertiary educated (27.9%, [*n* = 79/283] vs. 20.1%, [*n* = 67/333]; *p* = 0.0234) compared to those of integrated NCD-HTS. Standard of care HTS had significantly less proportions of females (53.9%, [*n* = 153/285] vs. 67.6%, [*n* = 225/333]; *p* = 0.0005), Zulu speakers (41.0%, [*n* = 116/283] vs. 49.0%, [*n* = 163/333]; *p* = 0.0480) and clients reporting their parents as their source of income (13.0%, [*n* = 37/284] vs. 20.5%, [*n* = 68/331]; *p* = 0.0135) as compared to integrated NCD-HTS (Table 1).

**Table 1:** PHRU Zazi Evaluation Study Participant Demographics by Phase (Phase 1, Feb-June 2018; Phase 2, June 2018–March 2019)

Variable	Overall	Phase 1	Phase 2	***P***-Value
**No. of enrolment**	617	284	333	
**Age (in years)**				
18–24 (%)	113 (18.4)	44 (15.5)	69 (20.7)	0.0680
25–34 (%)	223 (36.1)	95 (33.5)	128 (38.4)	
35–44 (%)	16 (26.7)	83 (29.2)	82 (24.6)	
≥ 45 (%)	116 (18.8)	62/ (21.8)	54 (16.3)	
Median (IQR)	33.0 (26.0–41)	35.0 (28.0–43)	31.0 (25.0–40)	0.0003
Mean (SD)	34.9 (10.8)	36.5 (10.9)	33.6 (10.5)	0.0008
Min, Max	(18–73)	(18–73)	(18–64)	
**Sex**				
Female (%)	378 (61.3)	153 (53.9)	225 (67.6)	0.0005
Male (%)	239 (38.7)	131 (46.1)	108 (32.4)	
**Race**				
Black (%)	612 (99.2)	281 (98.9)	331 (99.4)	0.6657
Coloured/mixed race (%)	5 (0.8)	3 (1.1)	2 (0.6)	
**Nationality**^**a**^				
South African (%)	592 (96.6)	268 (95.7)	324 (97.3)	0.2831
Other (%)	21 (3.4)	12 (4.3)	9 (2.7)	
**Ethnic group**^**a**^				
Zulu (%)	27 (45.3)	116 (41.0)	163 (48.9)	0.3544
Sotho (%)	101 (16.4)	50 (17.7)	51 (15.3)	
Tsonga (%)	70 (11.4)	31 (11.0)	39 (11.7)	
Tswana (%)	39 (6.2)	22 (7.7)	17 (5.2)	
Xhosa (%)	49 (8.0)	24 (8.5)	25 (7.5)	
Other (%)	78 (12.7)	40 (14.1)	38 (11.4)	
**Marital status**^**a**^				
Divorced/Widowed (%)	27 (4.4)	19 (6.7)	8 (2.4)	0.0298
Living together/Married (%)	153 (24.9)	66 (23.4)	87 (26.2)	
Single (%)	434 (70.7)	197 (69.9)	237 (71.4)	
**Highest education**^**a**^				
Up To High School (%)	247 (40.1)	111 (39.2)	136 (40.8)	0.0598
Matriculated (%)	223 (36.2)	93 (32.9)	130 (39.0)	
Tertiary education (%)	146 (23.7)	79 (27.9)	67 (20.2)	
**Source of money to live on?**^**a**^				
Employed (%)	227 (36.9)	111 (39.1)	116 (35.0)	0.0395
Parents (%)	105 (17.1)	37 (13.0)	68 (20.5)	
Pension (%)	18 (2.9)	12 (4.2)	6 (1.9)	
Self-employed (%)	118 (19.2)	61 (21.5)	57 (17.2)	
Social/Disability grant (%)	74 (12.0)	29 (10.2)	45 (13.6)	
Unemployed (%)	73 (11.9)	34 (12.0)	39 (11.8)	
**Wealth quintile**				
Quintile 1 (%)	163 (26.4)	69 (24.3)	94 (28.2)	0.2145
Quintile 2 (%)	138 (22.4)	73 (25.7)	65 (19.5)	
Quintile 3 (%)	164 (26.6)	74 (26.1)	90 (27.0)	
Quintile 4 (%)	124 (20.1)	52 (18.3)	72 (21.6)	
Quintile 5 (%)	28 (4.5)	16 (5.6)	12 (3.7)	

*IQR* interquartile range, *SD* standard deviation

^a^Overall may not be equal to the sample size as a result of missing responses

Of the 333 Phase 2 clients who completed a survey, 10.2% (*n* = 34/333) went on to become repeat clients within Phase 2.

### Reasons for declining HTS services for each HTS model

Only two clients opted out of HTS services in standard of care HTS, with one client dismissing CD4 count testing as she preferred to have it conducted at her ART referral clinic and another client declining being screened for STI symptoms as she responded that she was currently abstinent. A total of 36 survey responders (10.8% of clients) reported opting out of services in integrated NCD-HTS. Among these, 40% (*n* = 16/40, all women) refused cervical cancer screening for reasons related to reproductive health (i.e.; menstruation [*n* = 13/40, 32.5%]; having had a hysterectomy and therefore no cervix present [*n* = 2/40, 5.0%]; and sharing, *‘[I am] No longer interested in having babies’* [*n* = 1/40, 2.5%], which also represents a misunderstanding surrounding the purpose of the Pap smear procedure). Other reasons included the desire to reschedule their clinic visit (*n* = 10/40, 25.0%); being unprepared for or unsure of the new screenings (*n* = 6/40, 15.0%) (specifically for the pelvic exam); lack of time (*n* = 4/40, 10.0%), including either needing to return to work or needing to leave due to family obligations; worried about potential results (*n* = 2/40, 5.0%); having recently had a Pap smear or currently having an STI (*n* = 1/40, 2.5%, each).

### Client satisfaction with pre-test and post-test counselling by HTS model

**Fig. 2:**
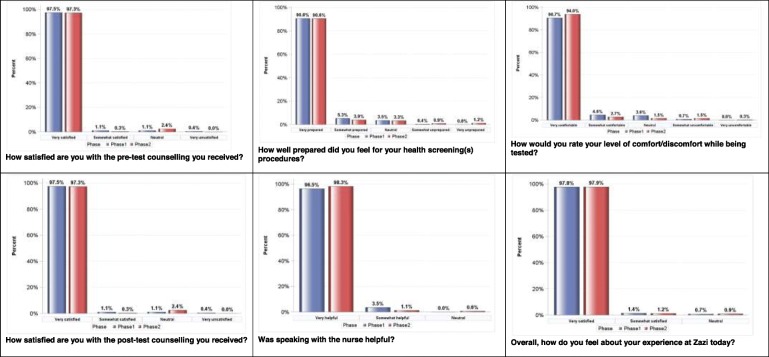
PHRU Zazi Evaluation Study Client Satisfaction by Phase (Phase 1, Feb-June 2018; Phase 2, June 2018–March 2019). **a** (top left): How satisfied are you with the pre-test counselling you received?. **b** (top middle): How well prepared did you feel for your health screening(s) procedures?. **c** (top right): How would you rate your level of comfort/discomfort while being tested?. **d** (bottom left): How satisfied are you with the post-test counselling you received?. **e** (bottom middle): Was speaking with the nurse helpful?. **f** (bottom right): Overall, how do you feel about your experience at Zazi today?

Figure 2 presents the distribution of client satisfaction by HTS model. The majority of participants were very satisfied with the pre-test and post-test counselling they received in standard of care HTS (97.5%,[*n* = 276/283] and 95.0%, [*n* = 268/282]) and integrated NCD-HTS (97.3%, [*n* = 322/331] and 96.7%, [*n* = 320/331]).

There were four main suggestions from seven standard of care HTS responders on how to improve pre-test counselling: minimise the paperwork (*n* = 3/7), provide in-depth information on health and/or disease (*n* = 2/7), explain the rapid testing technology and screening machines used (HIV test kits and BP cuff and monitor) (*n* = 1/7), and strengthen communication (*n* = 1/7), presumably around the rapid testing process and instruments used. Four responders in integrated NCD-HTS suggested different improvements for pre-test counselling. They were providing further health information, inclusive of ‘*relationship goals and health practice’*; creating set appointments times between ‘*8*–*15 h’*; providing additional screenings, such as ‘*prostate cancer*’; and explaining the new rapid testing machines (glucometer and lipidometer).

Only one standard of care HTS client shared a suggestion to improve post-test counselling, which was for the counsellor to provide ‘*tips on what the client should work towards’*. Presumably this relates to strengthening risk reduction counselling.

### Client satisfaction with health screening procedures by HTS model

Most participants were very prepared for the health screening procedures in standard of care HTS (90.8%, [*n* = 256/282]) and integrated NCD-HTS (90.6%, [*n* = 300/331]); and very comfortable with the level of comfort/discomfort while being tested in standard of care HTS (90.7%, [*n* = 254/280]) and integrated NCD-HTS (94.0%, [*n* = 311/331]). In standard of care HTS, of those who saw the nurse, 96.5% (*n* = 82/85) found speaking with the nurse very helpful, while 98.3% (*n* = 172/175) did in integrated NCD-HTS. Overall, the majority of participants were very satisfied with the experience they had at Zazi in both standard of care HTS (97.8%, [*n* = 272/278]) and integrated NCD-HTS (97.9%, [*n* = 320/327]). (Fig. 2).

Only ten standard of care HTS responders (3.5% of clients, [*n* = 10/284]) made suggestions to improve health screening procedures. Three responders desired additional health screenings such as ‘*STI test and blood sugar test*’ and also requested there to be less paperwork/administrative tasks; and one individual suggested each of the following: offer circumcision, improve the current testing procedure, explain the data collection process, and provide home visits. Integrated NCD-HTS contained only four responders (*n* = 4/333), of which three suggested additional integrated HTS to include breast cancer and eye screening; and one client requested standardised appointments for certain screenings for which one might be unprepared (e.g.; Pap smear).

### Clinic flow time and time-related client satisfaction by HTS model

**Fig. 3:**
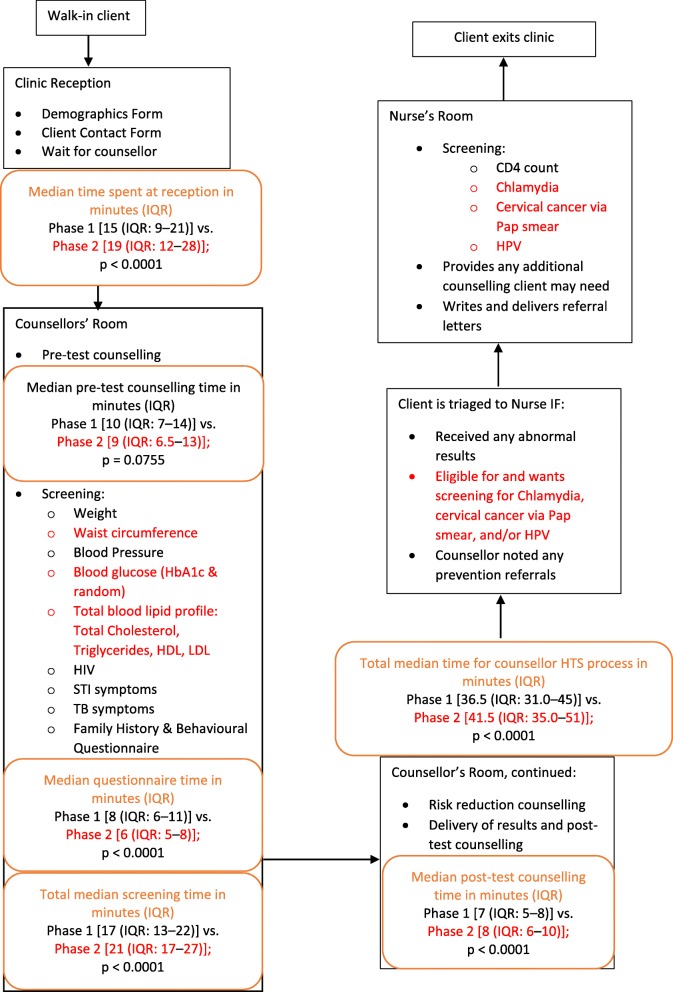
PHRU Zazi Evaluation Study HTS Clinic Time Flow Chart by Phase (Phase 1, Feb-June 2018; Phase 2, June 2018–March 2019). IQR, interquartile range; HbA1c, glycated haemoglobin/average blood glucose; HDL, high-density lipoprotein; LDL, low-density lipoprotein; STI, sexually transmitted infections; TB, tuberculosis; HPV, human papilloma virus. Black text denotes Phase 1 activities; black and red text denote Phase 2 activities. Orange text reports median clinic flow time in minutes

Participants in standard of care HTS took a significantly shorter median time (in minutes) in the counsellor-led HTS process relative to those in integrated NCD-HTS (36.5, IQR: 31.0–45.0, [*n* = 284/284] vs. 41.5, IQR: 35.0–51.0, [*n* = 332/333]; *p* < 0.0001) minutes (Table 2, Fig. 3). The majority of participants in standard of care HTS (93.2%, [*n* = 262/281]) and integrated NCD-HTS (94.9%, [*n* = 313/330]) were very/somewhat satisfied with the time spent at reception; though the median time in minutes was significantly shorter in standard of care HTS relative to integrated NCD-HTS (15.0, IQR: 9.0–21.0, [*n* = 265/284] vs. 19.0, IQR: 12.0–28.0, [*n* = 331/333]; *p* < 0.0001). Overall, participants felt very/somewhat satisfied by the amount of time spent on pre-test counselling (97.6% [*n* = 596/611]). The median time taken to conduct screenings (17.0, IQR: 13.0–22.0, [*n* = 281/284] vs. 21.0, IQR: 17.0–27.0, [*n* = 332/333]; *p* < 0.0001) and for post-test counselling (7.0, IQR: 5.0–8.0, [*n* = 284/284] vs. 8.0, IQR: 6.0–10.0, [*n* = 332/333]; *p* < 0.0001) was significantly shorter in standard of care HTS relative to integrated NCD-HTS. (Fig. 3).

**Table 2:** PHRU Zazi Evaluation Study Client Satisfaction with Clinic Flow Time by Phase (Phase 1, Feb-June 2018; Phase 2, June 2018–March 2019)

Variable	Overall	Phase 1	Phase 2	***P***-Value
**Total time (in minutes) in clinic**^**a**^				
Median (IQR)	*n* = 614, 96.0 (79.0–121)	*n* = 283, 86.0 (72.0–108)	*n* = 331, 102 (87.0–136)	< 0.0001
**Time (in minutes) spent in Reception**^**a**^				
Median (IQR)	*n* = 596, 17.0 (11.0–25)	*n* = 265, 15.0 (9.00–21)	*n* = 331, 19.0 (12.0–28)	< 0.0001
**Time (in minutes) for pre-test counselling**^**a**^				
Median (IQR)	*n* = 615, 9.00 (7.0–13.0)	*n* = 283, 10.0 (7.0–14.0)	*n* = 332, 9.0 (6.5–13.0)	0.0755
Min, Max	2, 83	2, 74	2,83	
**Total time (in minutes) for counsellor-led screenings**^**a**^				
Median (IQR)	*n* = 613, 19.0 (15.0–24.00)	*n* = 281, 17.0 (13.0–22.00)	*n* = 332, 21.0 (17.0–27)	<.0001
**Time (in minutes) for Family History and Behaviour Survey**^**a**^				
Median number of time (IQR)	*n* = 611, 7.0 (5.0–10)	*n* = 281, 8.0 (6.0–11)	*n* = 330, 6.0 (5.0–8)	<.0001
Mean number of time (SD)	*n* = 611, 8.1 (4.54)	*n* = 281, 9.4 (5.3)	*n* = 330, 7.1 (3.4)	<.0001
Min, Max number of time	*n* = 611, (2–65)	*n* = 281, (3–65)	*n* = 330, (2–22)	
**Time (in minutes) for post-test counselling**^**a**^				
Median (IQR)	*n* = 616, 7.0 (5.0–9)	*n* = 284, 7.0 (5.0–8)	*n* = 332, 8.00 (6.0–10)	<.0001
Min, Max	1, 41	1, 17	1,41	
**Total time (in minutes) for counsellor HTS process**^**a**^				
Median number of time (IQR)	*n* = 616, 39.0 (32.0–48.5)	*n* = 284, 36.5 (31.0–45)	*n* = 332, 41.5 (35.0–51)	<.0001
Mean number of time (SD)	*n* = 616, 43.1 (15.9)	*n* = 284, 39.2 (12.2)	*n* = 332, 46.4 (17.8)	<.0001
Min, Max number of time	*n* = 616, (18–138)	*n* = 284, (18–111)	*n* = 332, (21–138)	
**How would you rate the amount of time it took from when you entered the clinic until you were seen by a counsellor?**^**a**^				
I feel very/somewhat satisfied (%)	575 (94.1)	262 (93.2)	313 (94.8)	0.6820
I feel very/somewhat unsatisfied (%)	12 (2.0)	6 (2.1)	6 (1.8)	
Neutral (%)	24 (3.9)	13 (4.6)	11 (3.3)	
**How would you rate the amount of time it took the counsellor to provide the pre-test counselling you received?**^**a**^				
I feel very/somewhat satisfied (%)	596 (97.5)	270 (96.4)	326 (98.5)	–
I feel very/somewhat unsatisfied (%)	3 (0.5)	3 (1.1)	0 (0.0)	
Neutral (%)	12 (2.0)	7 (2.5)	5 (1.5)	
**How would you rate the amount of time it took the counsellor to provide the health screening tests and test results to you?**^**a**^				
I feel very/somewhat satisfied (%)	594 (97.2)	272 (97.1)	322 (97.3)	0.9264
I feel very/somewhat unsatisfied (%)	5 (0.8)	2 (0.7)	3 (0.9)	
Neutral (%)	12 (2.0)	6 (2.1)	6 (1.8)	
**How would you rate the amount of time it took the counsellor to provide the test results and post-test counselling you received?**^**a**^				
I feel very/somewhat satisfied (%)	594 (97.9)	273 (98.2)	321 (97.6)	–
I feel very unsatisfied (%)	1 (0.2)	1 (0.4)	0 (0.0)	
Neutral (%)	12 (2.0)	4 (1.4)	8 (2.4)	
**How would you rate the amount of time you spent with the nurse for further consultation and referrals?**^**a**^				
I didn’t see the nurse (%)	201 (43.7)	195 (69.9)	6 (3.3)	<.0001
I feel very/somewhat satisfied (%)	257 (55.9)	83 (29.7)	174 (96.1)	
Neutral (%)	2 (0.4)	1 (0.4)	1 (0.6)	

*IQR* interquartile range, *SD* standard deviation

^a^Overall may not be equal to the sample size as a result of missing responses

Counsellor-led HTS per phase is inclusive of pre-test counselling, phase-inclusive screenings, and post-test counselli

### Factors associated with total time (in minutes) spent in the clinic

Controlling for sex, living together/married (est = 6.548; *p* = 0.0467), number of tests a client underwent (est = 3.922; *p* < 0.0001), and higher overall satisfaction score (est = 1.210; *p* = 0.0201) were associated with longer time spent in the clinic. Those who matriculated spent less time in the clinic (est = − 7.250; *p* = 0.0253) (Supplementary Table 1).

### What clients liked by HTS model

There were 246 (86.6%, [*n* = 246/284]) responders depicting what clients liked about the clinic in standard of care HTS. Of these, 49.6% (*n* = 132/266) reported that they enjoyed the services and/or customer care they received. Two responders stated, ‘*Good services, as I am a regular participant*’ and ‘*Good service I got from reception to end’*. Additionally, 12.8% (*n* = 34/266) stated they liked the friendly and supportive staff they interacted with, and 9.4% (*n* = 25/266) shared they appreciated the clinic provider communication style. For example, one client shared, ‘*I was treated with respect and the staff was very friendly and kind. They explained to me everything and answered all my questions satisfactorily*.’ Other responses depicted clients liked feeling comfortable (6.4%, [*n* = 17/266]), the in-depth and professional counselling session (4.1%, [*n* = 11/266]), and the warm and welcoming clinic environment (3.4%, [*n* = 9/266]).

In integrated NCD-HTS, 307 (92.2%, [*n* = 307/333]) clients shared what they liked about ZAZI. The majority of responses shared that of Phase 1 responses – 35.5% (*n* = 124/349) appreciated the service, specifically the new screenings; 22.3% (*n* = 78/349) enjoyed the welcoming, professional and supportive staff; and 8.6% (*n* = 30/349) liked the effective communication of and information shared by the health care providers. One client responded, ‘*The staff member are very patient and explain things very well.’*

### What clients disliked by HTS model

Of the 206 (72.5%, [*n* = 206/284]) responders for standard of care HTS, 91.3% (*n* = 190/208) shared they did not dislike anything about Zazi. Of the remaining responders, the most common reasons for dissatisfaction were with the amount of time spent waiting in the clinic (2.9%, [*n* = 6/208]) and administration time spent during the clinic visit (i.e.; client registration systems and paperwork; 2.4%, [*n* = 5/208]). Other reasons for dissatisfaction were that treatment (1%, *n* = 2/208]), contraceptives (0.5%, [*n* = 1/208]), and snacks (0.5%, *n* = 1/208]) were not offered onsite; and outcome of results (unacceptance of one’s HIV diagnosis), wanting additional screenings, and untidiness of the reception area (0.5%, [*n* = 1/208] each).

Of the 300 (90.1%, [*n* = 300/333]) responders for integrated NCD-HTS, 88.7% (*n* = 268/302) disliked nothing. Of the remaining responses (*n* = 34/302), the top two reasons for dissatisfaction were duration of the clinic visit (55.9%, [*n* = 19/34]), with responses being ‘*I spent more time than expected*’ and *‘[I] waited for too long at reception’*; and the amount of administrative paperwork conducted (17.7%, [*n* = 6/34]).

### What clients wish ZAZI would have provided

When integrated NCD-HTS clients were asked to share if they wished the clinic would have provided them anything else, 23.7% (*n* = 79/333) made suggestions. Of these responders, 45.6% (*n* = 36/79) wished medications – ART, and for both chronic conditions and over-the-counter – were available onsite. Additionally, 20.3% (*n* = 16/79) requested additional services (e.g.; prostate and breast cancer screening, regular follow-up calls, and home visits for terminally ill patients); and 16.5% (*n* = 13/79) would have liked refreshments served. Only 7.6% (*n* = 6/79) clients wished the clinic could minimise time spent waiting.

## Discussion

This study reports a response of strong satisfaction from adult clients attending an integrated NCD-HTS clinic in Soweto, South Africa, regarding the counsellor-led HTS process and overall HTS experience, and with the time spent in clinic. Clients were appreciative of the newly integrated screenings and associated communication, and the welcoming clinic environment. Compared to standard of care HTS, including four additional counsellor-led NCD screening services (abdominal girth measurements, BMI categorizations, and blood cholesterol and glucose rapid testing) only increased the time of counsellor-led HTS by 5 min, on average. Clients who were married/living together, underwent more screenings, and had a higher overall satisfaction score spent significantly longer time within the clinic, while those who completed high school spent significantly less time in the clinic as compared to those who did not. While our study population demographics are similar to the general population of Soweto, increasing the likelihood of generalisability, the transferability of such an integrated HTS model, especially inclusive of nurse-led endocervical screenings, into the public sector would need further investigation.

Our study’s integrated NCD-HTS model has shown a high level of client satisfaction with healthcare delivery system factors, such as the warm and welcoming environment, inclusive of professional and non-judgemental healthcare providers; screenings offered; communication of health information; and clinic flow time. This conflicts with other studies’ findings that at the systems level, there are major gaps in safety, prevention, integration and continuity of care [29]. One in three patients across LMICs cited negative experiences with their health system in relation to attention, respect, communication, and length of visit – often shorter than 5 min; and less than one-quarter of people in LMICs believe their health system works well [29]. Specifically, as healthcare systems evolve to meet the growing demands associated with the NCD epidemic, there is potential for HIV-related stigma to influence where and how NCD care is provided for PLHIV and HIV-uninfected individuals [42, 43]. An emerging theme of a scoping literature review was that negative perceptions of healthcare providers – or fear of being mistreated due to stigma [44] - within HIV platforms may have a negative impact on the engagement with NCD care, either located within or outside of HIV platforms, and therefore effect the NCD-care continuum [42]. Stigma is reported as being an increasing barrier to woman accessing cervical cancer screening, due to fear of the diagnoses’ association with HIV [45]. While our results did corroborate with the other study’s conclusion of overall satisfaction being associated with longer length of visit, over one in ten of our study participants were repeat clients in the short-term, which constitutes positive user experience. Furthermore, the great majority of all clients opted in to each NCD screening integrated within our HTS platform, including cervical cancer screening for women.

Regarding clinic flow time, historically HTS is conducted in about 45 min – 10 min for pre-test counselling, 15–20 min to conduct the HIV rapid testing, and 15 min for post-test counselling [46]. The South African National HTS guidelines state that intensive and lengthy individual risk assessments during the pre-test counselling session is no longer needed or recommended [22]. Many providers in government health facilities simply do not have the time to perform detailed risk assessments due to the large number of clients. With the objective of PICT being timely detection of HIV and subsequent initiation of treatment, pre-test information can be simplified and shared through either individual or group educational outreach sessions, as long as they are presented in an age-appropriate manner [22, 47]. Though group sessions could decrease the HTS pre-test counselling time, group dynamics remain a challenge [46]. Post-test counselling may include individual risk assessments and risk reduction plans dependent on HIV status [47].

Zazi conducted individual - not group - pre-test counselling, and incorporated individual risk assessments and risk reduction plans into its post-test counselling as per national guidelines. While it took the counsellors a significantly longer time to conduct the rapid health screenings and post-test counselling in integrated NCD-HTS as compared to standard of care HTS, both HTS models of the counsellor-led HTS process were completed in a faster median time than the typical 45 min HTS timeframe. This was largely due to the faster than average post-test counselling timeframes. The shorter post-test counselling sessions could reflect the eventual triage to the nurse for clients needing referrals and further counselling. More than 96% of clients in each HTS model responded they were ‘*very/somewhat satisfied’* with the time it took to pass through the HTS process, and the large majority were ‘*very satisfied’* with the entire way it was conducted.

Given the relatively small difference in time spent between the counsellor-led standard of care HTS and integrated NCD-HTS models, despite introducing four new NCD-precursor screening measures, it is important to consider investigating if the same quality of care standard is actually met – in both screening conduct and information-sharing. Some studies have shown there is high satisfaction with healthcare among LMICs, despite widespread quality deficits, potentially due to low expectations and users either lacking knowledge about what constitutes good quality or being resigned about the quality of available services [48]. A study has shown that high-reported patient satisfaction likely overstates the quality of health services due to positively framing statements, which induces bias. Evaluations including patient satisfaction surveys need to complement patient satisfaction measures with more objective measures of quality [49]. Perhaps our quality of counselling did suffer, as there were shorter than average counselling sessions and clients suggested more robust information sharing, such as explaining the new rapid testing machines and providing further health practice information. However, our clients’ overall satisfaction score and increased number of screenings was associated with spending significantly more time in the clinic. This could potentially be attributable to the additional time spent providing client requested health information. Our clients of lesser education (i.e.; who had not yet matriculated) spent significantly more time in the clinic potentially due to longer discussions around health education.

With the addition of three, nurse-led endocervical screenings and increased client triage to the nurse to include NCD-referrals, the total median clinic time would increase even further and would likely push the integrated NCD-HTS clinic flow beyond the average HTS timeframe of 45 min. However, being an HTS clinic embedded within a research unit, there were also data collection aspects of Zazi that would not be implemented in government clinics, thus decreasing time spent. Clients shared their dislike for the time it took to complete such forms. For example, an extensive demographics form and client contact information sheet would not be distributed and completed in the waiting room, and screening would not include a lengthy family history and behavioural survey (median time of 8 and 6 min, for standard of care HTS and integrated NCD-HTS, respectively).

While the total integrated clinic time for integrated NCD-HTS was manageable for Zazi clients, it may not be possible for the public sector to scale up the same model of integrated services in a way that ensures the same level of positive user experience without additional human resources (i.e.; lay counsellors). Government clinics already have a heavy influx of patients and long queues. A qualitative study in South Africa reported as long as nurses are expected to manage large number of patients each day in primary care, HIV and AIDS services are unlikely to be successfully integrated into PHC, as inadequate staffing and healthcare facility space will negatively impact service delivery [50]. This is especially true in countries like South Africa, which are implementing Universal Test and Treat and same-day initiation policies. However, a case study that integrated NCD screening and care services within an existing HIV programme – similar to Zazi’s model - conducted in rural Malawi reported an improvement in access to NCD care for HIV-infected patients. The healthcare model’s success was largely attributable to strong leadership, allowing for real-time discussions about challenges encountered and allowing for process improvement, staff ownership of their specific duties, and a well-defined and uniform patient flow process. However, it was stated a missing element was a patient satisfaction survey to track the impact of the clinic’s redesign [51]. Furthermore, four studies examining the effects of various integrated models of care on HIV stigma have concluded some level of integration could potentially result in HIV-stigma reduction for PLHIV through combining care for HIV-uninfected NCD clients and PLHIV within the same facility [52–55].

In order to be scaled up within the public sector, the integrated NCD-HTS clinic model would have to utilise minimal paperwork/questionnaires in its clinic visit process, and employ enough clinic staff to support the client burden. Additionally, there would need to be clinic and staffing adjustments to accommodate women who desire cervical cancer screening, but through a scheduled appointment. Ideally, the healthcare staff conducting the rapid screenings would be well trained and confidently relied upon to conduct the testing, interpret findings, disseminate the results, and provide referrals without micromanagement. This would eliminate the need for and additional time spent to cross-check results and recommended referrals with a nurse or clinician, as was the process at Zazi. Due to the human resource shortfalls of health care practitioners, the public sector could replicate Zazi’s counsellor-led integrated NCD-HTS model. At the very least, the integration of rapid blood glucose and cholesterol testing into standard of care HTS would be feasible, as the time ranged between an acceptable 35 and 51 min. If Pap smears and HPV screening are to be integrated for female patients to reap the health benefits, additional data on added time spent would need to be analysed.

### Limitations

Most of the data captured in the Demographics Form, Client Contact forms, and Family History and Behavioural Questionnaire is affiliated with the PHRU Zazi HTS programme/study and not part of routine public healthcare. For clients either self-administering or being assisted with the completion of the Demographics and Client Contact Forms at reception, the time spent at reception was lengthened. The completion of the Family History and Behavioural Questionnaire increased the time it took for the counsellors to conduct the HTS screenings.

The study unfortunately did not collect clinic flow time in a standardised manner for the pre-registration time, the time it took the counsellors to QA/QC all counsellor-led screening results with the nurse, or the time the clients spent with the nurse, and therefore, did not include it within this paper. Therefore, a timeframe is not provided for the endocervical screenings for women (Chlamydia rapid test, and HPV and cervical cancer screenings), writing of referral letters and any further counselling conducted by the nurse. Furthermore, there may be a social desirability bias affect clinic flow times between HTS models due to the Hawthorne effect (i.e.; clinic staff knowing they are being timed).

A limitation of the Client Clinic Exit Survey and its content thematic analysis is the interpretativism - the researchers’ interpretation of the client response through their own understanding pair with thematic context. We tried to limit this by having multiple investigators agree on the analysis. The sample size of client responses per free-response question was also limited to how many clients actually chose to answer the question, rather than leave it blank. The five-point Likert response format was also subjective, in that the responses ‘*Very satisfied’*, ‘*Somewhat satisfied’*, etc. were not defined. Whereas not all variables had complete responses, the majority of participants responded with more than 98% response rate.

Additionally within integrated NCD-HTS, the Zazi clinic was assisting with HIV rapid test screening for the enrolment of women into HIV prevention trials conducted at PHRU. The higher volumes of females seen could be more of an intervention than by chance.

Despite the limitations, our data were reassuring, as we had limited missing data.

## Conclusions

This study has shown that it is possible to integrate counsellor-led NCD rapid testing into standard of care HTS, yielding client satisfaction. While counsellor-led integrated NCD-HTS significantly increases time to the clinic flow, as compared to standard of care HTS, it is still within historical HTS timeframes. It is suggested that rapid blood cholesterol and blood glucose testing be integrated into the standard of care HTS within the public sector to extend prevention efforts and quickly identify otherwise undiagnosed pre-cursors of NCD, as well as HIV-comorbidities. Studies regarding the transferability of these screenings into the public sector are required. While cervical cancer and HPV screenings were greatly appreciated by integrated NCD-HTS clients, further evidence on the impact to HTS clinic flow is needed to determine if such a robust integrated screening could be scaled up within the public sector.

## Supplementary information

**Additional file 1: Supplementary Figure 1.** Participant Flow Chart. This figure details the participant sampling.

**Additional file 2: Supplementary Table 1.** Factors associated with total time (in minutes) spent in ZAZI clinic. This table contains the data from both univariate and multivariate models which were run to determine the significant variables which were associated with the total time spent in clinic.

## Data Availability

The datasets used and/or analysed during the current study are available from the corresponding author on reasonable request.

## Abbreviations

ART: Antiretroviral therapy
BMI: Body mass index
BP: Blood pressure
CDC: Centers for Disease Control and Prevention
CVD: Cardiovascular disease
HbA1c: Glycated haemoglobin / average blood glucose
HCT: HIV counselling & testing
HPV: Human papilloma virus
HTS: HIV testing services
IQR: Interquartile range
NCDs: Non-communicable diseases
NDoH: South African National Department of Health
PCA: Principal Component Analysis
PHRU: Perinatal HIV Research Unit
PICT: Provider-initiated counselling & testing
PLHIV: People living with HIV
REDCap: Research Electronic Data Capture
STIs: Sexually transmitted infections
TB: Tuberculosis
UNAIDS: United Nations Joint Programme on HIV/AIDS
Wits HREC: University of the Witwatersrand Human Research Ethics Committee
