# Supplementary material for: Level of adult client satisfaction with clinic flow time and services of an integrated non-communicable disease-HIV testing services clinic in Soweto, South Africa: a cross-sectional study
Source: BMC Health Serv Res. 2020 May 11;20:404. doi: 10.1186/s12913-020-05256-9 (PMC7212607; doi:10.1186/s12913-020-05256-9)
Supplement: Supplementary file 1 — Additional file 1: Supplementary Figure 1. Participant Flow Chart. This figure details the participant sampling. [file 12913_2020_5256_MOESM1_ESM.docx]

57 clients from Phase 1 were also included in Phase 2. These clients were excluded in analysis for Phase 2.

617 client’s data were analysed for client satisfaction (Phase 1, n=284; Phase 2, n=333).

32 non-consenting clients for Phase 2 client clinic exit survey:

-27 referred from PHRU TB Clinic expedited out of Zazi

-2 did not understand study

-1 in a hurry

-1 not ready to participate in a study

-1 not willing to provide contact information for follow-up

41 non-consenting clients for Phase 1 client clinic exit survey:

-34 referred from PHRU TB Clinic expedited out of Zazi

-4 not interested in study

-2 in a hurry

-1 did not understand study

422 clients consented to Phase 2 health screenings and for their data being collected and used in aggregate analysis

325 clients consented to Phase 1 health screenings and for their data being collected and used in aggregate analysis

390 clients in Phase 2 consented to Zazi Client Exit

Survey

674 clients consented to Zazi Client Exit survey

(Phase 1 & 2)

284 clients in Phase 1 consented to Zazi Client Exit Survey
