# Supplementary material for: Level of adult client satisfaction with clinic flow time and services of an integrated non-communicable disease-HIV testing services clinic in Soweto, South Africa: a cross-sectional study
Source: BMC Health Serv Res. 2020 May 11;20:404. doi: 10.1186/s12913-020-05256-9 (PMC7212607; doi:10.1186/s12913-020-05256-9)
Supplement: Supplementary file 2 — Additional file 2: Supplementary Table 1. Factors associated with total time (in minutes) spent in ZAZI clinic. This table contains the data from both univariate and multivariate models which were run to determine the significant variables which were associated with the total time spent in clinic. [file 12913_2020_5256_MOESM2_ESM.docx]

**Supplementary Table 1: Factors associated with total time (in minutes) spent in ZAZI clinic**

|  | **Univariate** | | **Multivariate** | |
| --- | --- | --- | --- | --- |
| **Variable** | **Est (SE)** | **P-Value** | **Est (SE)** | **P-Value** |
|  |  |  |  |  |
| **Age (in years)** | -0.026 (0.134) | 0.8492 | - | - |
|  |  |  |  |  |
| **Gender** |  |  |  |  |
| Female vs. Male | 3.910 (2.958) | 0.1867 | -0.633 (3.081) | 0.8373 |
|  |  |  |  |  |
| **Marital Status** |  |  |  |  |
| Divorced/widowed vs. Single | -3.775 (7.080) | 0.5941 | -2.728 (6.814) | 0.6901 |
| Living together/married vs. Single | 7.809 (3.373) | 0.0210 | 6.548 (3.285) | **0.0467** |
|  |  |  |  |  |
| **Highest education** |  |  |  |  |
| Matriculated vs. Up To High School | -5.279 (3.304) | 0.1106 | -7.250 (3.233) | **0.0253** |
| Tertiary education vs. Up To High School | -6.974 (3.737) | 0.0625 | -4.591 (3.645) | 0.2083 |
|  |  |  |  |  |
| **Number of tests done** | 4.497 (0.602) | <.0001 | 3.922 (0.686) | **<.0001** |
|  |  |  |  |  |
| **Overall satisfaction score** | 2.060 (0.453) | <.0001 | 1.210 (0.519) | **0.0201** |
|  |  |  |  |  |

Est (SE)= Estimates (Standard Error)
